# Why Dentists Do Not Read

**Published:** 1903-04

**Authors:** Eugene S. Talbot


### WHY DENTISTS DO NOT READ.

BY EUGENE S. TALBOT, M.D., D.D.S.

In an address at the Pedagogic Society dinner, December, 1902, Dr. G. V. Black deplored the fact that dentists do not read. He feelingly urged the teachers of the various schools to stimulate their students to take journals, to buy books, and to read them.

It is notoriously the fact that there are fewer readers among dentists than any other profession. This has been recognized by editors of journals and others for many years. Why is it?

The most glaring illustration of this fact is that, with few exceptions, in papers written by dentists credit is not given to previous writers for the ideas they express. New ideas in a limited specialty like dentistry cannot be obtained without original research.

Dr. L. D. Shepard [1] asks, "Seriously, as a profession, do we read enough? Are we up to the times as a learned profession in the great throes of thought, science, and progress? One grand curse upon our profession from its beginning to the present day has been the lamentable and very general ignorance of its members." According to S. W. Lakin,[2] "education of the dentists should not only be coequal with the general practitioner, but should greatly exceed his in anatomy, physiology, pathology, therapeutics, and chemistry. It is quite impossible to keep abreast of the times in this inventive, progressive age without being familiar with the current dental literature. Dentists do not, as a rule, read an article a second or a third time." The duties of the dentist are said to be so laborious that he is too tired at night to think or read. Dental students are recruited from the ranks of the less cultured through the country dental office. Therefore he is much inferior to students who enter other professions. Neither of these explanations is satisfactory. Readers and scientists are the busiest men in the profession, and progressive men of all professions have been country-bred.

Dental literature antecedent to 1840 is that of highly-educated men. Books and papers showed thought and learning. They are equal to the best at that time and at the present day. These men were medically educated. Their work evinced vast reading in collateral sciences. Dental literature of value proceeds from men who possess other degrees than D.D.S. Here seems to be the solution of the problem. Take a dozen boys of like education, training, surroundings, and habits. Six enter medical schools, six dental schools. One-half are trained along broad lines with broad ideas in general medicine, while the dental students, although per-

---

[1] Dental Cosmos, December, 1865, vol. vii. p. 225.

[2] Ohio State Journal of Dental Science, vol. v. p. 560. Read before the Central Illinois Dental Society, October, 1885.

suing the same studies, are led to believe that, after all, tooth carpentering is *par excellence* an ideal in the profession of dentistry. It is only necessary to obtain enough knowledge of general subjects to get through college. This system of training may make dental students good mechanics, but certainly cannot make scientists.

The faulty methods of teaching the student in dental departments eventually turn out men certainly far below par in scientific training.

Let us hope that with an increasing college term students will be educated in general and special pathology, that scientific, not mechanical, dentistry will be dominant.

